# Peripheral neuropathy in HIV-infected children attending care and treatment clinic, at Muhimbili National Hospital, Dar es Salaam: a cross sectional study

**DOI:** 10.1186/s12883-021-02335-0

**Published:** 2021-08-13

**Authors:** Insiyah A Amiji, Helga E Naburi, Edward Kija, Livin P Mumburi

**Affiliations:** 1 Department of Pediatrics and Child Health, Muhimbili University of Health and Allied Sciences, Dar es Salaam, Tanzania; 2 Muhimbili National Hospital, Dar es Salaam, Tanzania

**Keywords:** Peripheral Neuropathy, HIV, Children, Care and Treatment

## Abstract

**Background:**

Peripheral neuropathy (PN) is a neurological complication of untreated Human Immunodeficiency Virus (HIV) infection or exposure to certain antiretroviral drugs. In Tanzania where HIV is a major public health problem, the burden of HIV associated peripheral neuropathy has not yet been well defined in children.Thisstudy investigated the prevalence and associated factors for peripheral neuropathy among children living with HIV, attending Care and Treatment Clinic (CTC) at Muhimbili National Hospital (MNH).

**Materials and methods:**

A cross-sectional study was conducted among 383 HIV positive children aged 5 to 18 years at MNH, CTC in Dar es Salaam between October to December 2019. All participants provided written assent/consent. Structured questionnaires designed for this study was used to collect data and screening for peripheral neuropathy was done on each participant using the Pediatric modified Total Neuropathy Score (Ped m TNS) that includes subjective and objective assessment. A score of 5 or greater on the Ped m TNS was used to define peripheral neuropathy. Data analysis was done using SPSS Version 23.

**Results:**

The prevalence of peripheral neuropathy among HIV infected children was 14.1 % (95 % CI (10.8 − 18 %). Common neuropathic symptoms were numbness, tingling sensation, reduced ankle reflexes and reduced sensation to light touch and pain that was limited to the toes. Low CD4 cell count (OR = 12.21; 95 % CI3.75–39.66; *p* = 0.0001), high viral load (OR = 10.54; 95 % CI 3.19–34.77; *p* = 0.0001), ART regime containing NRTI plus PI (OR = 3.93; 95 % CI 1.43– 10.74; *p* = 0.01) and the last exposure to isoniazid more than 6 months ago (OR = 3.71; 95 % CI 1.57–8.77; *p* = 0.003) were independent predictors for peripheral neuropathy.

**Conclusion:**

Peripheral neuropathy is common among HIV infected children attending CTC at MNH and its frequency increases with advanced disease. The choice of ART regimen and other drugs for treating comorbid conditions should carefully be evaluated.

**Supplementary Information:**

The online version contains supplementary material available at 10.1186/s12883-021-02335-0.

## Background

Peripheral neuropathy (PN) is a frequent neurological complication of Human Immunodeficiency Virus (HIV).The incidence increases with advanced disease or severe immunosuppression. It can be a result of cytopathic effects of the virus or the neurotoxiceffects of certain antiretroviral medications that causes inflammatory mediated neuronal cell damage [1]. Though these mechanisms differ in etiopathogenesis, the disorder is clinically and physiologically indistinguishable. In low income countries HIV infected children are at a higher risk to develop neurological complications because they also face multiple comorbidities such as anemia, tuberculosis, syphilis, herpes, malnutrition and poor socio-economic status which further compound the pathology and complicate it’s management [2]. Clinically, there is no standard definition of diagnosing PN. Patients may be asymptomatic or can experience one or more of the following symptoms; pain, numbness, tingling, aching, burning sensation in a glove and stocking distribution, reduced ankle reflexes, decreased pinprick and vibration sensation in the distal lower extremities [1, 3, 4]. The gold standard for diagnosis of PN is electrophysiological tests or skin biopsy which is invasive, costly and requires a high level of expertise for its interpretation, hence it cannot be done routinely especially in a resource limited setting. Several clinical screening tools that are user friendly and cost effective have been developed over the past few years to screen for HIV associated PN [3, 5]. Currently, there is no approved treatment for PN, but several drugs have been suggested for symptomatic control of neuropathic pain such as anticonvulsants like gabapentin and pregabalin, topical capsaicin patches, antidepressants, and nonspecific analgesics such as nonsteroidal anti-inflammatory drugs and opioids. This study aims to evaluate the magnitude of PN among HIV infected children using a simple clinical screening tool. This will facilitate early detection of this condition so that timely interventions can be offered to prevent its progression to debilitating symptoms later in life.

## Materials and methods

A hospital based cross sectional study was conducted at HIV Care and Treatment Clinic (CTC) at Muhimbili National Hospital (MNH) in Dar es Salaam during the months of October to December 2019. Our study population comprised of all confirmed HIV positive children attending CTC from 5to 18 years of age who were verbally competent. Children with neurological impairment such as cerebral palsy, stroke or spinal cord pathologies and developmental delays that would interfere with neurological examination were excluded from the study. Children who met the set inclusion criteria were consecutively recruited into the study until the desired sample size was reached. Laboratory diagnosis of HIV infection at MNH in infants and children < 18 months is done by detection of viral nucleic acid (RNA or pro-viral DNA) or viral antigens (p24) and in > 18 months by detecting antibodies to HIV using rapid tests or Enzyme Immunoassays (EIA) according to Tanzanian National AIDS Control Program guidelines [6]. Data was recorded in a structured questionnaire designed for this study, which was pre-tested in a pilot study to ensure validity of the questions (Supplementary File 1).

Information regarding demographic characteristics, anthropometric measurements (body mass index (BMI) and height for age using WHO classification system) to determine nutritional status, HIV disease and drug information were obtained from the patient’s history and medical records available during enrollment. The Ped m TNS screening tool consists of subjective and objective assessment of sensory, motor, and autonomic domains of the peripheral nervous system together with a 5-part neurologic examination. This tool has been used to screen PN in children with chronic illness such as cancer and diabetes mellitus [7, 8]. Prior to the commencement of this study, pediatricians working at the CTC were trained to use the Ped m TNS by the neurologist to assess PN. The tool was first translated from English to Swahili and then back translated to English by independent translators. It was then pre-tested in a pilot study which was conducted at the Paediatric Neurology clinic on children who had PN who were askedto express how they experienced the symptoms in their own words which wasthen verified in the translated version. Words such as *tingling, burning* and *numbness* were added inthe Swahili version of the tool using the description of symptoms from the children with PN. The adjusted tool was thentested in both groups of children with PN and those without PN for the second time. From the results of the pilot study the tool had a sensitivity and specificity of 86.7 and 83.3 % respectively and a false positive and false negative rate was 16.7 and 13.3 % respectively. The internal consistency of the tool was assessed using Cronbach’salpha and item–total score correlations. The ped m TNS demonstrated acceptable internal consistency with no items scoring less than 0.3 on the corrected item total correlation and an overall Cronbach’salpha of 0.83.

After putting all the above into consideration and confirming that both groups understood the symptoms, the tool wasused in the field for screening for PN. Objective assessment was done to determine light touch and pain sensation that was elicited in different dermatomes in the distal extremities using 10 g Semmes Weinstein monofilament and Medipin (with patient’s eyes closed) respectively, if the subject responded to the stimuli then it was normal for that extremity. Otherwise, further testing was done in more proximal areas of the limb. Vibration sensation was elicited by 128 Hz tuning fork placed perpendicular to the bony prominences (fingers, wrist, elbows, toes, and ankles) the cut off was at least 10 s followed by testing for deep tendon reflexes. A score of 5 or greater on the Ped m TNS indicated PN [9] (Supplementary File 2).

The variables measured in this study were age, sex, BMI and height for age, hemoglobin levels, CD4 cell count, HIV viral load, WHO clinical stage, duration of HIV illness, duration and combination of Anti-retroviral Therapy (ART) regime, isoniazid exposure and cotrimoxazole prophylaxis. Data analysis was done using IBM Corp. Released 2015. IBM SPSS Statistics for Windows, Version 23.0. Armonk, NY: IBM Corp software. Continuous variables were presented as mean ± standard deviation or median (interquartile range) respectively and t - test was used to determine the differences between means and their significance. Mann-Whitney *U* test was used to compare continuous variable with non-parametric distribution. Categorical variables were expressed as frequencies and group differences were compared using chi -square and Fisher’s exact test. Logistic regression models were applied to determine the independent predictors of PN .Results were reported as odds ratios (ORs) and 95 % confidence intervals (CI), *p* values < 0.05 was considered statistically significant.

 This study was approved by the Muhimbili University of Health and Allied Sciences (MUHAS) Ethical committee of Research and Publication with ethical registration number DA.287/298/01A. All methods were carried out in accordance with relevant guidelines and regulations. Permission to conduct this study at the CTC at MNH was granted by hospitals’ respective research office. Written informed consent was sought from the caregiver (parents/guardian) of the child and a formal written assent was also obtained from all children seven years and above who had the capacity to understand at a level they could comprehend using Swahili language.

## Results

### Patient characteristics

**Fig. 1:**
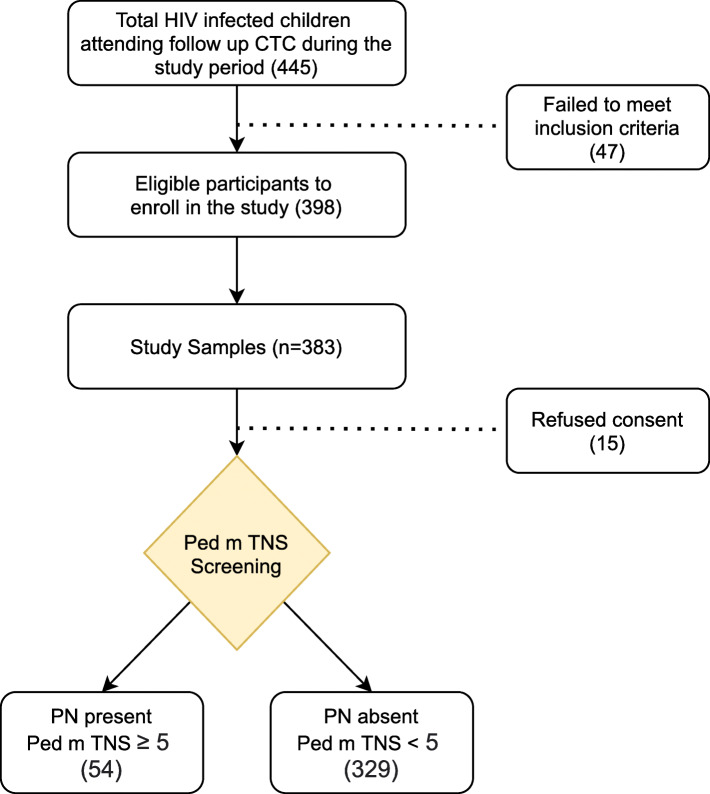
Patient recruitment

Four hundred and forty-five (445) HIV infected children attended CTC follow up clinic during the study period. Out of which three hundred and eighty-three (383) participants met the set inclusion criteria, gave consent, and were enrolled in the study 61 % (237/383) of who were males (Fig. 1). Majority of the participants had their BMI (59.5 %) and height for age (60.6 %) within normal range. The median duration of HIV infection was 12 years (IQR 8–16) and 67.6 % (259/383)were classified into WHO clinical disease stage 3 and 4. All participants were using ART during enrollment, 63.4 % (243/383) were on a regimen containing Nucleoside Reverse Transcriptase Inhibitor (NRTI) plus Dolutegravir (DTG). Out of the 383 participants,90.9 %(348/383) were prescribedisoniazid as part of Isoniazid Preventive Therapy (IPT) or anti-TB treatment during HIV-TB coinfection and 93.7 % (359/383) used cotrimoxazole prophylaxis as indicated according to the Tanzania national guidelines for management of HIV (Table 1).

**Table 1:** Distribution of demographic and clinical characteristic of 383 HIV infected children screened for PN

Variable	PN Present N (%)	PN Absent N (%)	Total *N* = 383 ( % )	*p- value
***Patient related factor***				
** Age (*years*)**				
5–9	8 ( 8.3 % )	88 ( 91.7 % )	95 ( 24.8 % )	0.9
10–14	18 ( 15. 5 % )	88 ( 84.5 %)	117 ( 30.5 % )	
15–18	28 (16. 4 % )	143 ( 83.6 % )	171 ( 44.6 % )	
** Sex**				
Male	33 ( 13.9 % )	204 ( 86.1 % )	237 ( 61.9 % )	0.02
Female	21 ( 14.4 % )	125 ( 85.6 % )	146 ( 38.1 % )	
** Mean Hemoglobin (*g/dl*)**	10.3 ± SD 1.4	10.5 ± SD 1.5		0.96 ^b^
** BMI (*kg/m***^***2***^***)***				
Normal	36 ( 15.8 % )	192 ( 84.2 % )	228 ( 59.5 % )	0.25
Underweight	18 ( 11.6 %)	137(88.4 %)	155 ( 40.5 % )	
** Height/Age**				
Normal	29 (12.5 %)	203 ( 87.5 %)	232 (60.6 %)	0.26
Stunting	25 (16.6 %)	126 (83.4 %)	151 (39.4 %)	
***Disease related factors***				
** WHO Clinical Stage**				
1 and 2	7 ( 5.6 %)	117 ( 94.4 %)	124 (32.4 %)	0.001
3 and 4	47 (18.1 %)	212 ( 81.9 %)	259 (67.6 %)	
** CD4 count (*cells/mm***^***3***^***)***				
< 350	22 ( 59.5 % )	15 ( 40.5 % )	37 ( 9.7 % )	0.0001
≥ 350	32 ( 9.2 % )	314 ( 90.8 % )	346 ( 90.3 % )	
** Viral load*(copies/ml)***				
≥ 1000	47 ( 38.8 % )	74 ( 61.2 % )	121 ( 31.6 % )	0.0001
< 1000	7 ( 2.7 % )	255 ( 97.3 % )	262 ( 68.4 % )	
** Median duration of HIV illness (*years*)**	13 (IQR 8–15)	12 (IQR 8–16)		0.66^a^
** Drug related factor**				
** Median duration of ARV use (*years)***	13 (IQR 8–15)	12 (IQR 8–16)		0.65^a^
** ARV drug combination groups**				
NRTI’s + NNRTI’s	7 ( 10.8 % )	58 ( 89.2 % )	65 (17 %)	0.02
NRTI’s + PI’s	18 ( 24 % )	57 ( 76 % )	75 ( 19.6 % )	
NNRTI’s + DTG	29 ( 11.9 % )	214 ( 88.1 % )	243 ( 63.4 % )	
** Isoniazid Exposure**				
Yes	54 ( 15 % )	305 ( 85 % )	359 ( 93.7 % )	0.02 ^c^
No	0	24 ( 100 %)	24 ( 6.3 % )	
** Last use of Isoniazid**				
Within 6 months	11 (8.9 %)	137 (92.6 %)	148 (38.6 %)	0.003
More than 6 months	43 (18.3 %)	216 (81.7 %)	259 (67.6 %)	
** Cotrimoxazole Prophylaxis**				
Yes	48 ( 14.4 %)	298 (85.6 %)	348 ( 90.9 % )	0.43 ^c^
No	4 ( 11.4 %)	31 (88.6 %)	35 ( 9.1 % )	

^a^Mann Whitney U test

^b^Independent samples t-test

^c^Fishers Exact Test

*P- value < 0.05 is significant

### HIV associated PN

The prevalence of PN in this study was 14.1 % (54/383) based on the assessment done using the Ped m-TNS (Fig. 1). The internal consistency of the tool was assessed using Cronbach’s alpha and item–total score correlations. Out of the participants who were symptomatic 14.4 % (21/146) were females and belonged to older age group of 15–18 years (16.4 %; 28/171). Participants who were symptomatic frequently reported sensory symptoms such as numbness (85.21 %; 46/54), tingling sensation (61.11 %; 33/54 ),reduced ankle reflexes (59.24 %; 32/54), reduced sensation to light touch (88.81 %; 48/54) and a few had loss of vibration sense (38.87 %; 21/ 54 ) that was limited to the finger and toes (Table 2). Furthermore, upon asking the participants if they experienced any functional symptoms from the screening tool, most reported they did not have any difficulties in execution of their daily activities.

**Table 2:** Prevalence of symptoms and signs in children with PN (*n *= 54)

	**N (%)**
**Symptoms**	
**Numbness**	46/54 ( 85.21 % )
**Burning Sensation**	7/54 ( 13.0 % )
**Tingling Sensation**	33/54 ( 61.11 % )
**Signs**	
**Ankle reflex Reduced**	32/54 (59.24 % )
**Light touch Reduced**	48/54 (88.81 % )
**Pain Sensation Reduced**	39/54 (72.22 % )
**Vibration sense Reduced**	21/54 (38.87 % )

The median duration of HIV infection among symptomatic patients was 13 (IQR 8–15) years and their CD4 counts were less than 350 cell/mm^3^ compared to their counterpart (59.5 % vs. 40.5 %, *p* = 0.0001). Nearly a quarter ( 24 %) of HIV positive children with PN used ART regime containing NRTI’s plus PI that include *Abacavir/ Lamivudine with Lopinavir/ritonavir* and *Zidovudine* /*Lamivudine with Lopinavir/ritonavir.* We also observed thatall symptomatic participants who had PN had a history of isoniazid exposure during their lifetime (Table 3).

**Table 3:** Univariate and multivariate analysis of factors associated with HIV - PN

Characteristic	Crude OR (95 % CI)	*p* –value	Adjusted OR (95 % CI)	*p-* value
***Patient related factors***				
** Age*(years)***				
5–9	1		1	
10–14	2.43 (0.97–6.07)	0.06	1.86 ( 0.54–6.36)	0.32
15–18	2.46 ( 1.03–5.87)	0.04	2.60 (0.74–9.10)	0.14
** Sex**				
Male	0.96 (0.53–1.73)	0.9	-	-
Female	1			
** Mean Hemoglobin*(g/dl)***	1.01 ( 0.84–1.19)	0.96	-	-
** BMI (*kg/m***^***2***^**)**				
Normal	1			
Underweight	0.7 ( 0.38–1.28)	0.25	-	-
** Height/Age**				
Normal	1			
Stunting	1.38 ( 0.77–2.47)	0.26	-	-
*** Disease related factors***				
** WHO Clinical stage**				
1 and 2	1		1	
3 and 4	3.7 (1.62– 8.46)	0.002	3.12 ( 0.83–11.69)	0.09
** CD4 counts*(cells/mm***^***3***^***)***				
< 350	14.4 ( 6.78–30.41)	0.0001	12.21 ( 3.75–39.6)	0.0001
≥ 350	1		1	
** Viral Load (copies/ml)**				
≥ 1000	23.13 ( 10.01–53.34 )	0.0001	10.54 (3.19–34.77)	0.0001
< 1000	1		1	
** Duration of HIV illness*( years)***	1.0 ( 0.95–1.07)	0.73	**-**	**-**
***Drug related factors***				
** ARV drug combinations**				
NRTI + NNRTI	0.89 ( 0.37–2.13)	0.79	0.91 ( 0.26–3.18)	0.89
NRTI + PI	2.33 ( 1.21–4.49)	0.01	3.93 (1.43–10.74)	0.01
NNRTI + DTG	1		1	
** Duration of ARV use*(years)***	1.01 ( 0.95–1.07)	0.72	-	**-**
** Isoniazid Exposure**				
Yes	0.01 ( 0 - ∞ )	0.99	-	-
No	1			
** Last exposure to Isoniazid**				
Within 6 months	1		1	
More than 6 months ago	2.78 (1.38–5.61)	0.004	3.71 (1.57–8.77)	0.003
** Cotrimoxazole Prophylaxis**				
Yes	0.76 (0.26–2.27)	0.63	-	**-**
No	1			

Possible factors that were observed to be associated with PN on univariate analysis were further analysed for confounding effect. Multivariate analysis was done for those factors and additionally others with *p* < 0.2 (Table 4). In the multivariate analysis model we found that low CD4 cell count (OR = 12.21; 95 % CI 3.75–39.66; *p* = 0.0001), high viral load (OR = 10.54; 95 % CI 3.19–34.77; *p* = 0.0001), ART regime containing NRTI plus PI (OR = 3.93; 95 % CI 1.43– 10.74; *p* = 0.01) and the last exposure to isoniazid more than 6 months ago (OR = 3.71; 95 % CI 1.57–8.77; *p* = 0.003) were independent predictors for PN (Table 4).

**Table 4:** Univariate and multivariate analysis of factors associated with HIV - PN

Characteristic	Crude OR (95% CI)	***p*** –value	Adjusted OR (95% CI)	***p-*** value
***Patient related factors***				
**Age*(years)***				
5-9	1		1	
10-14	2.43 (0.97- 6.07)	**0.06**	2.52 (0.74 – 8.56)	0.11
15-18	2.46 ( 1.03 – 5.87)	**0.04**	3.68 ( 1.08 – 12.49)	0.18
**Sex**				
Male	0.96 (0.53 – 1.73)	0.9	-	-
Female	1			
**Mean Hemoglobin*(g/dl)***	1.01 ( 0.84 – 1.19)	0.96	-	-
**BMI (*kg/m***^***2***^**)**				
Normal	1			
Underweight	0.7 ( 0.38 – 1.28)	0.25	-	-
**Height/Age**				
Normal	1			
Stunting	1.38 ( 0.77 – 2.47)	0.26	-	-
***Disease related factors***				
**CD4 counts*(cells/mm***^***3***^***)***				
< 200	14.4 ( 6.78 – 30.41)	**0.0001**	5.21 ( 2.0 – 13.57)	**0.001**
≥ 200	1		1	
**Viral Load (copies/ml)**				
< 1000	1		1	
≥ 1000	23.13 ( 10.01 -53.34 )	**0.0001**	26.31 (7.91 – 86.51)	**0.0001**
**WHO clinical stage**				
1 & 2	1		1	
3 & 4	3.7 ( 1.62 -8.46 )	**0.002**	0.43 (0.12 – 1.58)	0.21
**Duration of HIV illness*( years)***	1.0 ( 0.95 – 1.07)	0.73	**-**	**-**
***Drug related factors***				
**ARV drug combinations**				
NRTI + NNRTI	0.89 ( 0.37 – 2.13)	0.79	1.13 (0.32 – 4.01)	0.84
NRTI + PI	2.33 ( 1.21 – 4.49)	**0.01**	5.67 (2.11– 15.22)	**0.01**
NNRTI + DTG	1		1	
**Duration of ARV use*(years)***	1.01 ( 0.95 – 1.07)	0.72	-	**-**
**Isoniazid Exposure**				
Yes	0.01 ( 0 - ∞ )	0.99	-	-
No	1			
**Time interval since last use of Isoniazid**				
< 6 months	1		1	
≥ 6 months	2.78 (1.38 – 5.61)	**0.004**	4.54 (1.91-10.79)	**0.001**
**Cotrimoxazole Prophylaxis**				
Yes	0.76 (0.26 – 2.27)	0.63	-	**-**
No	1			

## Discussion

This study aimed to determine the prevalence of PN and associated factors among children living with HIV who attended CTC follow up clinic at MNH. Results from this study shows that the prevalence of PN in HIV positive children attending CTC at MNH was 14.1 %. The prevalence of HIV associated PN in children varies greatly among studies from different countries ranging from 13 to 34 % [10–13]. The prevalence from this study was consistent with a study done in HIV positive Peruvian children who were diagnosed using electrophysiological tests [14]. However, higher rates have been found in South African and Brazilian children who were diagnosed using clinical scoring tools like Neuropathy Symptom Score (NSS)/Neuropathy Disability Score (NDS) and electrophysiological studies respectively [10, 11, 15]. The variability in results could be attributed to the diversity of patient population and the various methods used to diagnose PN in different studies, where some clinicians used clinical scoring tools while others used electrophysiological studies. Despite the variation in the population demographics, the prevalence of PN obtained in HIV positive children using clinical scoring tool was almost similar to that obtained from electrophysiological studies [16]. Based on our findings there are possibilities that such children are not been identified in their routine clinic visits as they may fail to express their symptoms and no formal assessment for PN was done.

### Predictors of PN among HIV infected children

In this study the independent predictors of PN among HIV infected children attending CTC at MNH were severe immunosuppression as reflected by a low CD 4 count (< 350 cell/mm^3^), a high viral load (≥ 1000 copies/ml), use of NRTI’s plus PI’s and the last exposure to isoniazid more than 6 months ago. These factors have also been observed to be strong predictors of PN in both children and adults living with HIV in different settings [15, 17–19].

### Clinical presentation

Using the Pedm TNS screening tool sensory symptoms were frequently reported by participants than motor symptoms and most did not experience functional impairments that interfered with their daily activities. This could possibly indicate that sensory neuropathy was common in children with HIV infection in our study. These findings were comparable to a study done by Remco et al., who assessed HIV associated PN in children using a different clinical neuropathy screening tool [18]. In HIV positive adults in addition to the neuropathic symptoms patients have also been observed to have significant functional impairment like fatigue, slower gait, poor cognitive functioning and depression compared to those who did not have PN [19].

It has been highlighted in previous reports that many patients also have subclinical neuropathy during evaluation that was either co-incidentally detected by electrophysiology studies or during a skin biopsy [15]. This could also explain the difference in reporting the clinical presentation across studies since many patients could be asymptomatic at presentation, and would not meet the diagnostic criteria of a clinical scoring tool and be missed during a clinical evaluation. However, despite the limitations of the clinical scoring tools, in this study the tool identified children with PN who had never been diagnosed before. This shows its usefulness in a routine care settings where healthcare workers often depend on caregivers to subjectively report history of such symptoms. Thus, findings from this study emphasize a role of regular active assessment of PN for all HIV positive children attending CTC follow up clinic using a standard tool.

### Patient related factors

In this study PN was equally distributed among males and females. Floeter et al., reported that the risk of PN in HIV infected children was higher in older children above 13 years than younger ones [10]. Few adult studies have also shown association of PN with older and taller patients regardless of whether they had received ART [11]. When the two factors were combined neuropathy rates increased from 18 % in younger and shorter patients to 33 % in younger, taller patients, the cut off age being ≤ 40 years and height ≥ 170 cm [12]. However, the association of this combination with PN was not observed in this study. Furthermore, there was no significant difference in relation to sex and hemoglobin levels among those with or without PN. Patients with HIV infection of all age groups are exposed to nutritional deficiencies and it is a strong predictor for the progress of the disease, survival, and functioning in the course of illness. One study reported that children with malnutrition [14] had a higher risk to develop distal sensory PN than those who did not have malnutrition however, we observed no association between malnutrition and PN in this study. Neurological manifestation associated with micronutrient deficiencies such as vitamin B12 has been reported more in HIV positive adults than in children [20]. This could be explained by the fact that in our study very few children had malnutrition and those who had malnutrition did not have the severe forms.

### Disease related factors

The odds of developing PN were significantly high among children with severe immunosuppression as reflected by a low CD4 count < 350 cell/mm^3^ and a high viral load ≥ 1000 copies/ml in this study. Similar observation has been reported in earlier studies involving adults before ART became widely available [20, 21]. In contrast, few reports involving children and adults during ART era have not shown association between immunosuppression and advanced disease with PN despite several studies suggesting that low CD4 cell counts represents a risk factor for HIV neuropathy [14, 22, 23]. This could be due to early initiation of ART and other unstudied risk factors that may have attributed to PN observed in these population.

Majority of children enrolled in the current study were using ART’s, 90.3 % (346/383) had CD4 counts > 350cell/mm^3^ and 68.4 % (262/383 ) had achieved viral suppression, out of those only some were observed to have neuropathic symptoms, suggests a possibility of a subclinical PN that was not detected before initiation of ART as baseline screening is not routinely done in children attending CTC follow up clinics [15].

Patients with long standing HIV infection could theoretically be at greater risk for peripheral nerve damage. However, in this study there was no significant association observed between PN and the duration of HIV infection. Similar findings were also reported in previous studies involving children [17, 18]. This could be possibly be explained by the fact that most of HIV infection in children results from vertical transmission from mother to child, thus unlike adults time of diagnosis does not reflect the time of infection in this population. However, longer duration of HIV related systemic symptoms have been observed in adults with PN compared to those who did not have PN [22]. Possible risk factors for instance alcohol use, opportunistic infections, HIV related malignancies, prolonged exposure to neurotoxic medications and environmental exposures may at least partially account for the high neuropathy rates observed in adults with longer duration of HIV infection than in children [2, 15].

### Drug related factors

There was a significant association between the use ARTregimethat contained NRTI’s plus PI’s with PN. Previous studies have shown that some of the NRTI’s can cause PN in both adults and children, particularly the dideoxy-NRTI’s (d – drugs) like stavudine, didanosine, and zalcitabine which are known to cause mitochondrial damage in the peripheral nerves [13, 24, 25]. These drugs have been phased out from the ART regimens for both children and adults living with HIV, and none of the participants in our study were using ART regime containing d-drugs. Few studies involving adults also showed that the use of PI’s like indinavir, lopinavir and saquinavir in the ART regime was associated with PN [12]. However, the role of PI induced neuropathy is still unclear and the independent risk of neuropathy attributable to PI’s is likely to be small and should be outweighed by its vital role in viral suppression in the ART regimens [26]. Furthermore, drug induced toxic neuropathy is dose dependent and reversible compared to HIV associated PN thus patients on ART need a close evaluation.

There was significant association between participants reported their last exposure to isoniazid more than 6 months ago and PN. Isoniazid is commonly prescribed in Tanzania, either for HIV-TB co-infection and as part of IPT for duration of 6 months in children and adults. Majority of the participants attending CTC were prescribed isoniazid either currently or in the past at a dose of 10 mg/kg for 6 months. Isoniazid induced neuropathy is dose dependent and it has been extensively reported in literature [13, 24]. There is no clear demarcation on the dose and duration of onset of symptoms after initiating isoniazid. Isoniazid reduces the biological active pyridoxine (Vitamin B6) that regenerates and nourishes nerve cells. Thus it is recommended that patients using isoniazid must also receive pyridoxine supplements, to prevent development of isoniazid-induced peripheral neuritis [25]. Unfortunately, none of the participants in our study who were using isoniazid as part of IPT or TB treatment were receiving pyridoxine. Thus, the observed association between the use of isoniazid and occurrence of neuropathy in this study could be because the pyridoxine supplementation was not implemented as recommended.

Treatment PN is mostly symptomatic depending on the underlying cause. Several approaches have been used in adults to relieve these symptoms such as pain medication and physical therapy which can also be used in children to relieve the symptoms. Furthermore, in case of drug induced neuropathy early identification and stopping the offending ART medication can help to prevent neuropathy from getting worse.

This study had some limitations. Firstly, this was a cross sectional study limited by ability to establish a causal relationship. Secondly, HIV associated PN was based on a clinical diagnosis therefore its incidence may have been underestimated since electrophysiologic studies or skin biopsies were not performed. Furthermore, none of the participants had a baseline neuropathy screening done before initiating or changing ART which made it difficult to establish the attributing factor causing injury to the peripheral nerves whether it was HIV related or drug induced neurotoxicity. Lastly, other factors such vitamin B12 deficiency especially in malnourished children, diabetes and opportunistic infections that causes neurological complications in HIVinfectionwere not studied in this population. Despite the limitations this study has identified a neglected problem among HIV infected children, showing the importance of routine assessment for PN in HIV clinics especially in children with advance disease.

## Conclusions

HIV associated PN is common in Tanzanian children attending CTC at MNH. Advanced disease, use of ART regime containing NRTI’s and PI’s, and the last exposure to isoniazid more than 6 months ago were independent predictors for HIV associated PN in this study. We recommend that selection ofART regimen and other drugs for treating comorbid conditions should be carefully doneand pyridoxine supplementation should be given to children using isoniazid to prevent the development of PN.

## Supplementary Information

**Additional file 1. **Questionnaire: ENGLISH VERSION

**Additional file 2.** The Pediatric Modified Total Neuropathy Screening Tool ( English Version)

## Data Availability

The datasets used and analyzed during the current study are not publicly available because participants have not given consent for public availability of their data. However, the data are available from the corresponding author on reasonable request.

## Abbreviations

3TC: Lamivudine
ABC: Abacavir
AIDS: Acquired Immunodeficiency Syndrome
ART: Antiretroviral Therapy
AZT: Zidovudine
BMI: Basal Metabolic Rate
BPNS: Brief Peripheral Neuropathy Score
CD 4: Cluster of Differentiation
CHARTER: CNS HIV Anti-Retroviral Treatment Effects Research
CTC: Care and Treatment Clinic
DRG: Dorsal Root Ganglia
DTG: Dolutegravir
DNA: De-oxy ribonucleotide acid
EID: Early Infant Diagnosis
GABA _A_: Gamma Amino Butyric Acid type A
HAART: Highly Active Anti-Retroviral Therapy
HIV: Human Immunodeficiency Virus
IFN: Interferon Gamma
IL: Interleukin
IPT: Isoniazid Preventive Therapy
ISN: Isoniazid
LPV/R: Lopinavir/ritonavir
MNH: Muhimbili National Hospital
NDS: Neuropathy Disability Score
NNRTI: Non- Nucleoside Reverse Transcriptase Inhibitors
NRTI: Nucleoside Reverse Transcriptase Inhibitors
NSS: Neuropathy Symptom Score
Ped- m TNS: Pediatric- Modified Total Neuropathy Score
PEPFAR: President’s Emergency Plan for AIDS Relief
PI: Protease Inhibitor
PMTCT: Prevention of Mother to Child Transmission
RCH: Reproductive and Child Health
TDF: Tenofovir
TNF: Tumor Necrosis Factor
UNAIDS: Joint United Nations Program on HIV and AIDS
UNICEF: United Nations Children’s Emergency Fund
WHO: World Health Organization
