# Supplementary material for: Peripheral neuropathy in HIV-infected children attending care and treatment clinic, at Muhimbili National Hospital, Dar es Salaam: a cross sectional study
Source: BMC Neurol. 2021 Aug 13;21:314. doi: 10.1186/s12883-021-02335-0 (PMC8361625; doi:10.1186/s12883-021-02335-0)
Supplement: Supplementary file 1 — Additional file 1. Questionnaire: ENGLISH VERSION [file 12883_2021_2335_MOESM1_ESM.docx]

**TITLE: Peripheral Neuropathy in HIV-infected children attending Care and Treatment Clinic, at Muhimbili National Hospital, Dar es Salaam: A Cross sectional study**

**Authors**: Insiyah Amiji ^1^, Helga E Naburi ^1^, Edward Kija ^1^, Livin P Mumburi ^2^

**Authors Affiliations**

1. Department of Pediatrics and Child Health, Muhimbili University of Health and Allied Sciences, Dar es Salaam, Tanzania
2. Muhimbili National Hospital, Dar es Salaam, Tanzania

*Corresponding Author:* Dr. Insiyah A Amiji

[insiyah_amijee@hotmail.com](mailto:insiyah_amijee@hotmail.com)

**Supplementary File 1**

**Questionnaire: ENGLISH VERSION**

**Study Title: Peripheral Neuropathy in HIV-infected children attending Care and Treatment Clinic, at Muhimbili National Hospital, Dar es Salaam: A Cross sectional study**

Participant ID no………………………………………..

## **PART A**

## **SOCIODEMOGRAPHIC DATA**

1. AGE………………………………………………………………………….
2. SEX :

□ MALE □ FEMALE

1. CONTACT DETAILS……………………………………………………..
2. A family history of Peripheral neuropathy

□ YES □ NO

**ANTHROPOMETRIC MEASUREMENTS**

1. HEIGHT (M)………………………………………………………………...
2. WEIGHT (KG)………………………………………………………………
3. BMI (kg/m^2^)………………………………………………………………….

**HIV RELATED INFORMATION**

1. Care and Treatment Clinic file number………………………………………
2. Duration of illness (years)…………………………………………………….
3. Current CD 4 COUNT (last 6 months) ………………………………………

□ ≥ 350 cells/mm^3^

□ < 350 cells/mm^3^

1. Current VIRAL LOAD (last 6 months) ……………………………

- < 1000 copies/ml
- ≥ 1000 copies/ml

1. WHO clinical stage…………………………………………………… ………
2. Is the participant receiving antiretroviral treatment: (if yes go to Qn 15 and 16)

□ YES □ NO

1. Duration of antiretroviral regime………………………………………………

□ < 6 months □ ≥ 6 months

1. Combination of Antiretroviral Treatment used ( ART)…….………………….
2. Opportunistic infections currently on treatment or in the past?

………………………………………………

………………………………………………

………………………………………………

………………………………………………

1. Is the participant receiving Cotrimoxazole prophylaxis as recommended?

□ YES

□ NO

1. Does the participant have history of isoniazid exposure (as part of IPT or treatment for TB in the past 6 months): if yes go to Qn 20

□ Yes

□ No

1. Duration of Isoniazid exposure

□ < 6 months

□ ≥ 6 months

20. Hemoglobin levels in the last visit ( within 6 months) ………………
