# Supplementary material for: Peripheral neuropathy in HIV-infected children attending care and treatment clinic, at Muhimbili National Hospital, Dar es Salaam: a cross sectional study
Source: BMC Neurol. 2021 Aug 13;21:314. doi: 10.1186/s12883-021-02335-0 (PMC8361625; doi:10.1186/s12883-021-02335-0)
Supplement: Supplementary file 2 — Additional file 2. The Pediatric Modified Total Neuropathy Screening Tool ( English Version) [file 12883_2021_2335_MOESM2_ESM.docx]

**TITLE: Peripheral Neuropathy in HIV-infected children attending Care and Treatment Clinic, at Muhimbili National Hospital, Dar es Salaam: A Cross sectional study**

**Authors**: Insiyah Amiji ^1^, Helga E Naburi ^1^, Edward Kija ^1^, Livin P Mumburi ^2^

**Authors Affiliations**

1. Department of Pediatrics and Child Health, Muhimbili University of Health and Allied Sciences, Dar es Salaam, Tanzania
2. Muhimbili National Hospital, Dar es Salaam, Tanzania

*Corresponding Author:* Dr. Insiyah A Amiji

[insiyah_amijee@hotmail.com](mailto:insiyah_amijee@hotmail.com)

**Supplementary File 2**

**The Pediatric Modified Total Neuropathy Screening Tool ( English Version)**

Sensory Symptoms: ______ (record worst score for the three sensations)

“ Do you have any parts of your body that are tingly, numb (can hardly feel), or burning (like fire)?” ______ Tingly ______ Numb ______ Burning (record number for each) If yes, “Where you have those feelings?”

0 None

1 Symptoms limited to fingers or toes

2 Symptoms extend to ankles or wrists

3 Symptoms extend to knee or elbow

4 Symptoms above knee or elbow

Functional Symptoms: ______ (record worst score of the three questions)

“Do you have trouble buttoning shirts or tying shoes (zipping zippers if more age appropriate)?”

“Do you have trouble walking such as tripping frequently?” ________

“Do you have trouble going up or downstairs?” _______

If yes to any, “Is it…..(read choices)” and record after each question

0 Not Difficult

1 A little difficult

2 Somewhat difficult

3 I need help

4 I can’t do that at all

Autonomic Symptoms: _____ (record worst score of the two questions)

“Do you feel dizzy or light-headed when you get up out of bed?”______

“Do your hands or feet feel hotter or colder than normal?” ______

0 Never

1 A little bit

2 Sometimes

3 Very much

4 Almost always

**Clinical Testing:**

Light Touch Sensation: ______

0 Normal

1 Reduced in fingers/toes

2 Reduced up to wrist/ankle

3 Reduced up to elbow/knee

4 Reduce to above elbow/knee

Pin Sensibility: _____

0 Normal

1 Reduced in fingers/toes

2 Reduced up to wrist/ankle

3 Reduced up to elbow/knee

4 Reduce to above elbow/knee

Vibration Sensibility: _______ (worst score)

0 Normal

1 Reduced in fingers/toes

2 Reduced up to wrist/ankle

3 Reduced up to elbow/knee

4 Reduce to above elbow/knee

Strength: _____ Worst Score (Score R / L)

Level: Great Toe ___/___ ankle DF___/___ finger abd___/___ wrist ext___/___

0 Normal

1 Mild weakness

2 Moderate weakness

3 Severe weakness

4 Paralysis

DTR: _____ (Achilles, Patellar)

0 Normal

1 Ankle reflex reduced

2 Ankle reflex absent

3 Ankle reflex absent, others reduced

1. All reflexes absent

Total Score: ­­­­­­­­­­_____/ 32
